# Sporadic Early-Onset Colorectal Cancer Is a Specific Sub-Type of Cancer: A Morphological, Molecular and Genetics Study

**DOI:** 10.1371/journal.pone.0103159

**Published:** 2014-08-01

**Authors:** Sylvain Kirzin, Laetitia Marisa, Rosine Guimbaud, Aurélien De Reynies, Michèle Legrain, Pierre Laurent-Puig, Pierre Cordelier, Bernard Pradère, Delphine Bonnet, Fabienne Meggetto, Guillaume Portier, Pierre Brousset, Janick Selves

**Affiliations:** 1 Centre de Recherche en Cancérologie de Toulouse, Unité Mixte de Recherche, 1037 INSERM – Université Toulouse III, Toulouse, France; 2 Department of Surgery, Centre Hospitalier Universitaire de Toulouse, Toulouse, France; 3 “Cartes d'Identité des Tumeurs” Program, Ligue Nationale Contre le Cancer, Paris, France; 4 Department of Oncology, Centre Hospitalier Universitaire de Toulouse, Toulouse, France; 5 Laboratoire de Biochimie Biologie Moléculaire, Hôpitaux Universitaires de Hautepierre, Strasbourg, France; 6 Bases Moléculaires de la réponse aux xénobiotiques, Université Paris Descartes, INSERM, UMR-S775, Paris, France; 7 Department of Pathology, Centre Hospitalier Universitaire de Toulouse, Toulouse, France; Singapore General Hospital, Singapore

## Abstract

Sporadic early onset colorectal carcinoma (EOCRC) which has by definition no identified hereditary predisposition is a growing problem that remains poorly understood. Molecular analysis could improve identification of distinct sub-types of colorectal cancers (CRC) with therapeutic implications and thus can help establish that sporadic EOCRC is a distinct entity. From 954 patients resected for CRC at our institution, 98 patients were selected. Patients aged 45–60 years were excluded to help define “young” and “old” groups. Thirty-nine cases of sporadic EOCRC (patients≤45 years with microsatellite stable tumors) were compared to both microsatellite stable tumors from older patients (36 cases, patients>60 years) and to groups of patients with microsatellite instability. Each group was tested for *TP53*, *KRAS*, *BRAF*, *PIK3CA* mutations and the presence of a methylator phenotype. Gene expression profiles were also used for pathway analysis. Compared to microsatellite stable CRC from old patients, sporadic EOCRC were characterized by distal location, frequent synchronous metastases and infrequent synchronous adenomas but did not have specific morphological characteristics. A familial history of CRC was more common in sporadic EOCRC patients despite a lack of identified hereditary conditions (p = 0.013). Genetic studies also showed the absence of *BRAF* mutations (p = 0.022) and the methylator phenotype (p = 0.005) in sporadic EOCRC compared to older patients. Gene expression analysis implicated key pathways such as Wnt/beta catenin, MAP Kinase, growth factor signaling (EGFR, HGF, PDGF) and the TNFR1 pathway in sporadic EOCRC. Wnt/beta catenin signaling activation was confirmed by aberrant nuclear beta catenin immunostaining (p = 0.01). This study strongly suggests that sporadic EOCRC is a distinct clinico-molecular entity presenting as a distal and aggressive disease associated with chromosome instability. Furthermore, several signaling pathways including the TNFR1 pathway have been identified as potential biomarkers for both the diagnosis and treatment of this disease.

## Introduction

Colorectal cancer (CRC) is most commonly seen in elderly adults with a median age at diagnosis of 70 years [1]. However, according to US registries the incidence of CRC in young adults is rising constantly, at a rate of 1.5% per year between 1992 and 2005 in adults aged 20–49 [2]. Early onset CRC (EOCRC) is an aggressive disease with poor differentiation and is classically located in the left colon. Individuals can be predisposed to EOCRC through heredity and inflammatory bowel diseases. The most well-defined hereditary forms of CRC are Lynch syndrome and familial adenomatous polyposis (FAP) that account for 2–4% and less than 1% of total CRC cases, respectively [3]. The relative contribution of inflammatory bowel diseases and FAP to EOCRC is modest since both conditions can easily be identified by their clinical features and managed by screening and prophylactic treatment according to established guidelines. In contrast, Lynch syndrome does not display a specific phenotype and frequently leads to carcinoma, making up one-third of EOCRC cases [4]. Thus, the majority of EOCRC are sporadic cases that lack genetic markers indicating predisposition.

CRC is a heterogeneous disease which is classically divided into three sub-types [5], according to the molecular mechanisms driving its transformation: (i) Chromosomal instability (CIN), the predominant mechanism, which is characterized by microsatellite stable tumors (MSS), loss of heterozygosity and major chromosomal changes in tumor-suppressor genes and oncogenes [9]; (ii) An epigenetic change known as the CpG island methylator phenotype (CIMP) which causes transcriptional silencing by methylation of CpG–rich regions in the promoter of tumor-suppressor genes. CIMP is responsible for the vast majority of sporadic MSI tumors through silencing of the promoter of the MMR gene *hMLH1* [7], [8]; (iii) Microsatellite instability (MSI), which is the hallmark of Lynch syndrome and is characterized by the accumulation of frame shift mutations in microsatellite sequences due to a deficiency in mismatch repair (MMR) genes [6]. However, this currently used molecular classification of CRC is based on only a few common DNA markers.

As yet the molecular markers of sporadic EOCRC have not been thoroughly explored and their individual contributions towards carcinogenesis have not been characterized. Genome expression profile (GEP) analysis could improve the molecular classification of CRC and help to identify clinical entities and biomarkers that could predict the response to treatment. We recently used this type of approach to identify six molecular sub-types of CRC that arise through distinct biological pathways [10].

The purpose of the present study was to investigate sporadic EOCRC using an integrated approach combining exhaustive clinico-pathological data with genetic analyses in order to determine the specific clinical features and molecular profiles of this entity. For this study we first identified a homogeneous group of sporadic EOCRC that was defined by having a cancer diagnosis at under 45 years of age, having no identified hereditary predisposition to CRC and having an MSS profile. We next compared specific genetic, epigenetic and gene expression data from this group with that from other well-defined groups of CRC.

## Materials and Methods

This study was designed to investigate sporadic EOCRC cases (MSS tumors from young patients) and compare them to counterpart MSS tumors from older patients. We also included CRC with MSI (both from young and old patients) since they constitute a well-characterized model of CRC oncogenesis.

### Patient and sample selection

**Figure 1:**
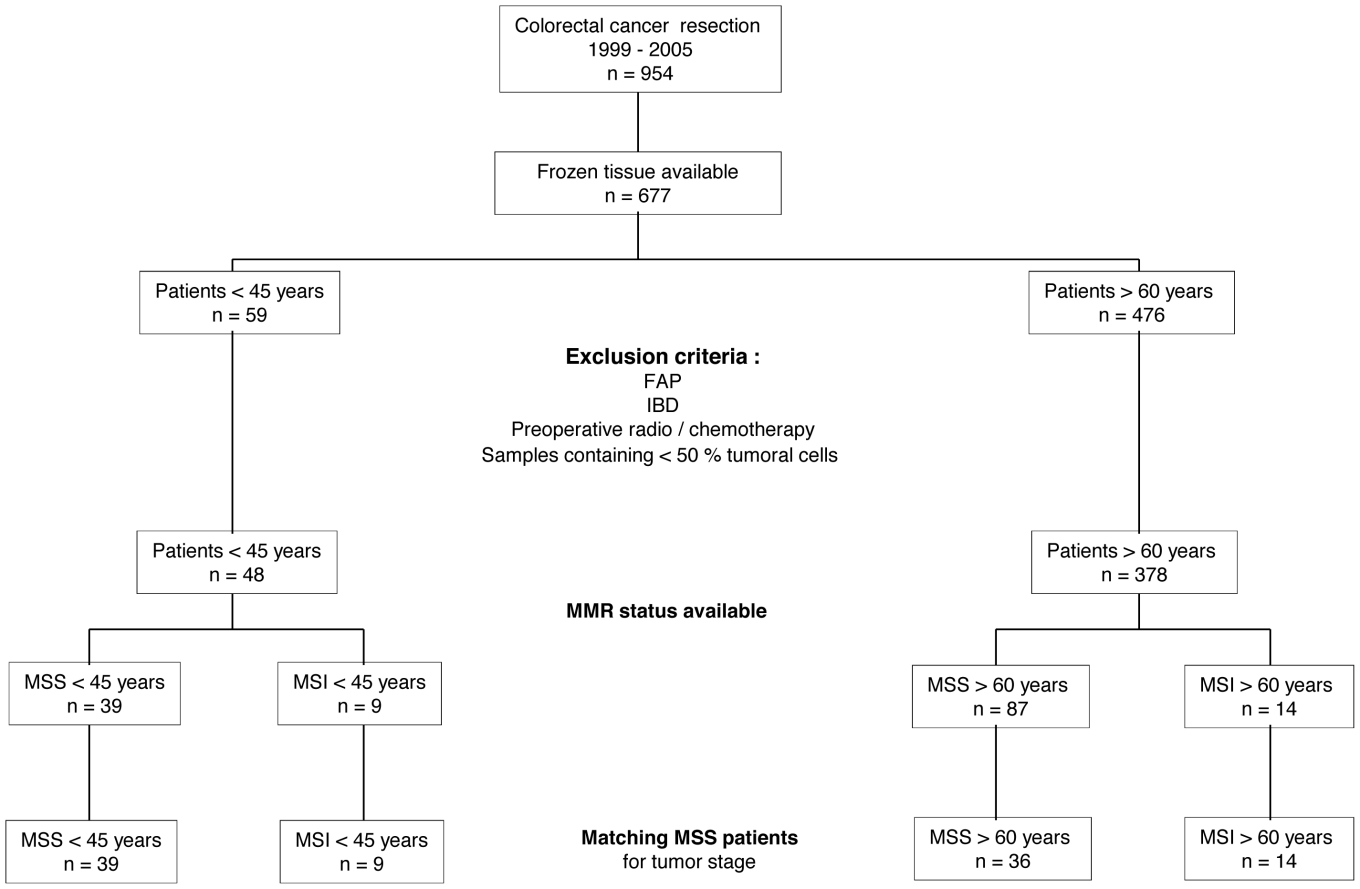
Consort diagram. Consort diagram depicting the selection of patients from a single institution, on the basis of available frozen tissue and after applying exclusion criteria. Four groups were defined by taking into account age and MMR status. The study was designed to allow the comparison of MSS early-onset CRC with other well-defined groups of CRC. FAP: Familial Adenomatous Polyposis. IBD: Inflammatory Bowel Disease. MMR status: Mismatch Repair status. MSS: Microsatellite stable. MSI: Microsatellite unstable.

Between April 1999 and December 2005, 954 patients were treated and followed-up for CRC at our institution. Patients under 45 years (from hereon referred to as “young” patients) and over 60 (“old”) were considered for comparison. Exclusion criteria were: unavailable frozen tissue; diagnosis of FAP or IBD; preoperative chemo/radiotherapy; samples containing less than 50% tumor cells; and unknown MMR status. Due to a high proportion of stage IV MSS tumors from young patients, MSS tumors from young and old patients were matched according to tumor stage. After sample selection, 98 patients were available for the study, as depicted in **Figure 1**. Four groups of patients were defined: MSS-Y (MSS-tumor<45 years), 39 cases; MSI-Y (MSI tumor<45 years), 9 cases; MSS-O (MSS tumor>60 years), 36 cases; and MSI-O group (MSI tumor>60 years), 14 cases.

### Ethics Committee Approval

All patients were prospectively registered in a central secured database declared to the Commission Nationale Informatique et Liberté (1365952). The use of the collected tumors was approved by the Toulouse Hospital board (CRB-Cancer Toulouse, DC-2008-463, AC-2008-820,CPP2). Written informed consent was obtained from all subjects.

### Studied parameters

Seventy-nine variables of interest were extracted from the database and analyzed. These included clinical data (familial pedigree, cancer site, clinical presentation at diagnosis, treatment modalities, survival) and tumor characteristics (complete pathological data, presence of synchronous adenoma in the resected specimen, MMR status, presence of genetic and epigenetic alterations). Clinical follow-up of patients was performed according to published national guidelines [11]. Survival curves were established from the date of diagnosis according to Kaplan-Meier calculations. Details of studied variables are provided in **Table S1**.

### MMR status and familial pedigree

MMR status was determined at diagnosis (previous to the study) by assessing microsatellite instability status using a pentaplex PCR and by using immunohistochemistry (IHC) to test for the expression of MLH1, MSH2 and MSH6, as previously described [12]. Mismatch repair deficiencies (dMMR tumors) were defined by MSI and/or loss of expression of one of the MMR proteins, whereas proficient MMR (pMMR) tumors corresponded to MSI-low or MSS tumors expressing the MMR proteins.

The pedigree of all young patients was assessed by a single clinical geneticist (RG). Genetic testing for the 2 major MMR genes *hMLH1* and *hMSH2* was performed in the case of MSI tumors or when personal or family cancer history was present in young patients. The diagnosis of Lynch syndrome was formally confirmed by the identification of a deleterious germline mutation in an MMR gene. Cases of MSI tumors displaying an absence of MSH2 or MSH6 staining with IHC were considered as probable Lynch syndrome.

### Mutational analysis

Genotyping of key mutations occurring in colorectal cancer (*KRAS*, *BRAF*, *TP53* and *PI3KCA*) was performed following DNA extraction from frozen tissue. The seven most frequent mutations on codons 12 and 13 of *KRAS*, and the codon V600E mutation in *BRAF* were assessed by allelic hybridization using Taqman probes, as previously described [13]. Direct sequencing of *TP53* exons 5 to 9 and *PI3KCA* exons 9 and 20 was performed in parallel using the Sanger technique (Beckman Coulter, Danvers, MA, USA).

### Methylator phenotype assessment

CIMP profiles were determined using a panel of five markers as described by Weisenberger *et al*: *CACNA1G*, *IGF2*, *NEUROG1*, *RUNX3* and *SOCS1*. After DNA bisulfite treatment, two multiplex methylation-specific PCRs were performed. Fragment analysis was carried out by capillary electrophoresis on an automatic sequencer (Beckman Coulter, Danvers, MA, USA). Methylator phenotype-positive cases (CIMP+) had 3 or more methylated promoters while CIMP-negative ones had less than 2 methylated promoters, defined according to established criteria [14].

### RNA extraction and microarray analysis

Total RNA was isolated using Trizol (Invitrogen, Carlsbad, CA) and checked for purity, integrity and quantity. Samples were then amplified, labeled and hybridized on an Affymetrix Human Genome U133 plus2 GeneChip, following the manufacturer's one-cycle target labeling protocol (Affymetrix, Santa Clara, CA). The chips were scanned with an Affymetrix GeneChip Scanner 3000 and raw intensities were extracted from subsequent images using GCOS 1.4 software (Affymetrix). Data were normalized using the Robust Multiarray Averaging (RMA) method, implemented in the R package affy [15]. Data are available at the NCBI Gene Expression Omnibus repository (accession number: GSE39084).

### Unsupervised analysis

Class discovery was performed by consensus clustering of expression profiles, as previously described [16]. Probe sets were first filtered to keep those expressed (i.e. with a normalized intensity value over 15) in at least 10% of samples with a variance significantly higher than the median variance of all probe sets (p<0.01). Then, 7 lists were obtained which included 1% to 50% of the most variant probe sets (based on the robust coefficient of variation). For each probe set list, samples were clustered using 1-Pearson correlation for distance metric and Ward linkage, yielding 7 dendrograms. Partitions in k clusters (k from 2 to 8) were derived from each dendrogram and a consensus partition was calculated for each value of k, yielding 7 consensus partitions.

### Supervised analysis

Associations between clinical and molecular annotations and sample groups were tested for statistical significance using the Chi-squared test or Fisher exact test where appropriate. Genes differentially expressed between tumors from young and old MSS patients were selected based on Limma moderated t-test p-values adjusted for multiple testing by the Benjamini & Hochberg method (<0.05) [17]. Pathways from the Biocarta database were tested for enrichment of deregulated genes by combining the following methods: Globaltest (R package globaltest), SAM-GS, and Tuckey [18], [19]. The p-values of each method were converted into ranks and the mean rank was computed to order pathways.

### Beta catenin signaling pathway activation

Immunohistochemistry was performed on formol-fixed and paraffin-embedded blocks from the 98 patients of the study with the mouse monoclonal antibody beta catenin (Clone 14, BD BioSciences, dilution 1/200, Ventana XT autostainer (Tucson, AZ, USA). All slides were reviewed by two pathologists blinded to groups of patients (JS and SK). Activation of the beta catenin signaling pathway was defined by strong nuclear staining in over 50% of tumor cells, with or without diffuse cytoplasmic staining and with a loss of cell membrane staining [20].

## Results

### Clinico-pathological characteristics of sporadic EOCRC

The sporadic EOCRC group consisted of 39 patients, distributed as follows: 17 were 40–45 years old, 13 were 35–40 years, 5 were 30–35 years, 2 were 25–30 years and 2 were under 25 years of age. Such a distribution in age is in line with data published in the US registry [1]. In comparison with tumors from MSS old patients, sporadic EOCRC (MSS young patients) displayed distinctive features. Rectal tumors were more abundant in sporadic EOCRC (41% versus 8.3%, p = 7.4 ^e-04^) with less frequent adenomas seen on resected specimens (8% versus 33%, p = 0.013). Notably, 15 (38%) sporadic EOCRC patients had a family history (first- or second-degree relative) of CRC compared to 3 (9%) in the group of MSS old patients (p = 0.013). A pedigree analysis of these 15 patients revealed that 3 of them had a clear autosomal dominant transmission inheritance pattern with multiple family members affected. Two of the 3 were from the same family but no germline mutation has been identified to date. The remaining 12 patients had an undetermined mode of transmission with only one member of the ascendant family having CRC, usually at an advanced age (>60 years). In accordance with French recommendations, *MUTYH* mutation analysis was performed for only 1 patient who presented with 8 adenoma, but this analysis was negative [21]. A personal history of CRC was observed only in old patients (including both MSS and MSI tumors). All tumors from MSI young patients arose in the context of Lynch syndrome, and 3 patients (21.5%) from the MSI old group were also Lynch patients. When considering the entire study population, the other significant differences observed between groups were linked to MMR deficiency, namely more frequent proximal location (p = 2.0 ^e-05^), poor cell differentiation (p = 0.033) and frequent mucinous component (p = 0.012). The main characteristics of the studied population are reported in **Table 1**.

**Table 1:** Clinico-pathological features of each group showing significant differences among the 79 variables studied.

	MSS Young n = 39	MSS Old n = 36	MSI Young n = 9	MSI Old n = 14	p value	p value MSS	p value MSI
**Age** (years mean ± SD)	39.1±5.8	71.2±7.3	36.7±4	74.6±9.2	-	-	-
**Sex** M/F	16 (41%)/23 (59%)	24 (67%)/12 (33%)	5 (56%)/4 (44%)	7 (50%)/7 (50%)	0.17	0.046	0.87
**Tumor location**					2.0 ^e-05^	1.8^e-03^	0.11
Right colon	6 (15.4%)	17 (47.2%)	5 (55.6%)	13 (92.9%)			
Left colon	16 (41%)	16 (44.4%)	4 (44.4%)	1 (7.1%)			
Rectum	16 (41%)	3 (8.3%)	0	0			
Multiple	1 (2.6%)	0	0	0			
**Tumoral stage** (UICC)					-	-	-
0	2 (5%)	2 (5%)	0	0			
I	2 (5%)	2 (5%)	3 (33%)	1 (7%)			
II	11 (28%)	10 (28%)	1 (11%)	9 (64%)			
III	8 (21%)	8 (22%)	2 (22%)	4 (29%)			
IV	16 (41%)	14 (39%)	3 (33%)	0			
**Metastatic site**					0.02	0.026	-
Unique	7 (17.9%)	12 (33.3%)	3 (33.3%)	0			
Liver	6	6	2	0			
Peritoneum	1	5	1	0			
Lung	0	1	0	0			
Multiple	9 (23.1%)*	2 (5.6%)**	0	0			
**Presence of synchronous adenoma**	3 (8%)	12 (33%)	0	3 (21%)	0.015	0.013	0.39
**Cell differentiation**					0.033	0.64	0.82
Well – moderate	35 (95%)	32 (89%)	7 (78%)	9 (64%)			
Poor	2 (5%)	4 (11%)	2 (22%)	5 (36%)			
**Mucinous component**					0.012	0.74	0.62
Absent	21 (75%)	20 (62%)	2 (25%)	3 (21%)			
<10%	1 (4%)	4 (12%)	3 (38%)	2 (14%)			
10 – 50%	4 (14%)	6 (19%)	1 (12%)	3 (21%)			
>50%	1 (4%)	1 (3%)	2 (25%)	4 (29%)			
Presence without precision	1 (4%)	1 (3%)	0	2 (14%)			
**CRC antecedent**					0.057	0.013	0.47
Personnal	0	1 (3%)	0	2 (14%)			
First degree	4 (16%)	3 (9%)	2 (33%)	2 (14%)			
Second degree	11 (29%)	0	1 (11%)	2 (14%)			
**Lynch syndrome**					2.1 e ^-16^	0.73	0.001
No	39 (100%)	36 (100%)	0	11 (78. 5%)			
Yes	0	0	9 (77.8%)	3 (21.5%)			

Data are expressed as absolute number and relative percentage. SD: standard deviation, M/F: male/female, UICC: Union for International Cancer Control (2002 classification), CRC: colorectal cancer. *: One case with liver and ovary metastases, 6 cases with liver and lung metastases, one case with liver and brain metastasis and one case with ovary and peritoneum metastasis. **: Two cases presented with liver and peritoneum metastases.

**Figure 2:**
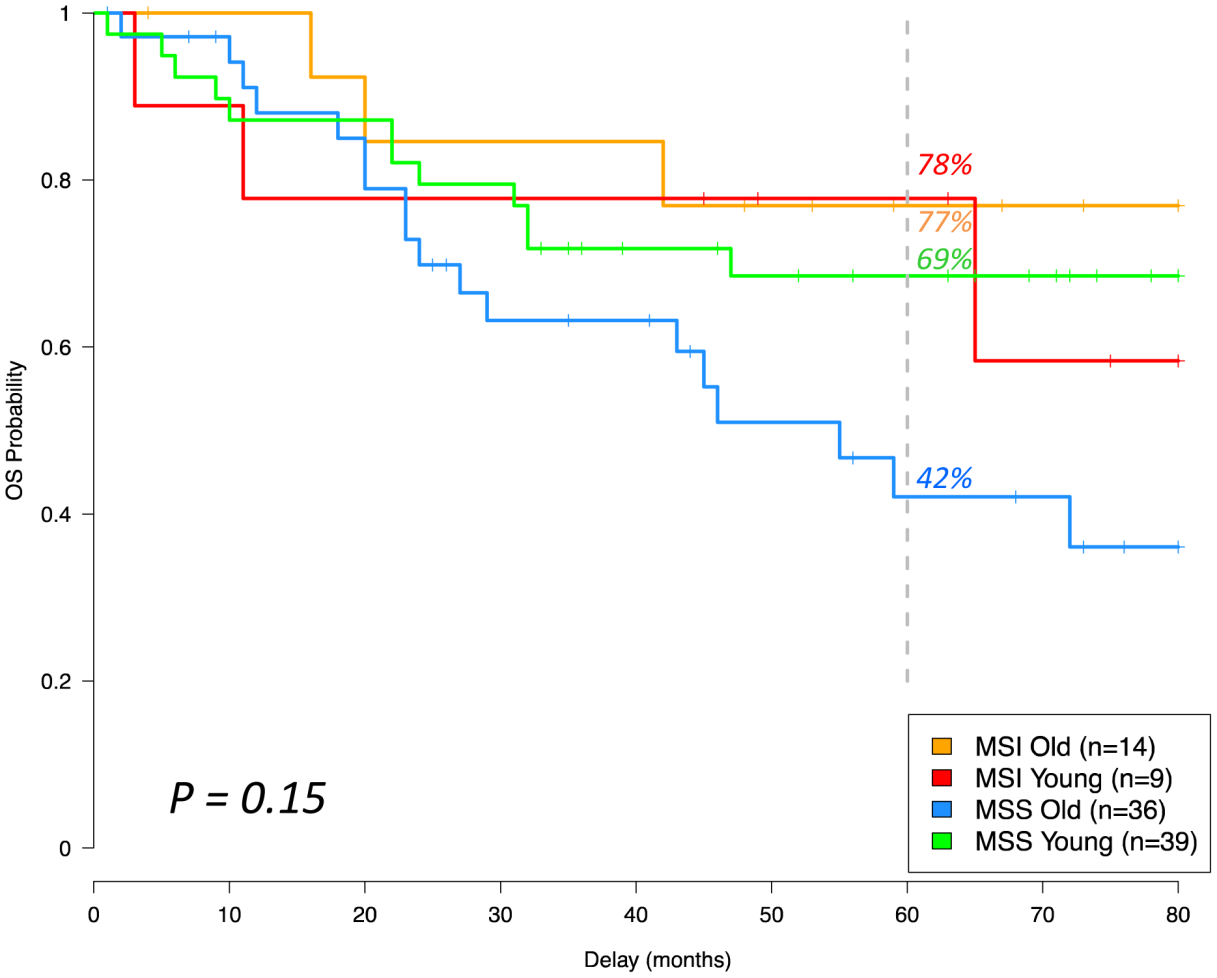
Overall survival in the four patient groups. Each group had a specific overall survival profile although none were significantly different from each other (p = 0.15). MSS: Microsatellite stable. MSI: Microsatellite unstable.

Sporadic EOCRC is an aggressive disease frequently presenting with metastases at diagnosis. The location of metastatic tumors was predominantly in the liver and lungs, as observed for CRC. A specific survival profile was observed for each group of patients, with overall survival better for MSI than MSS patients. Despite a high burden of metastases (with 23.1% versus 5.6% having multiple metastatic sites at diagnosis), sporadic EOCRC patients had a better 5-year overall survival than MSS old patients, although this was not significant (69% versus 42%, p = 0.09). The five year overall survival rate was roughly the same for MSI patients, although MSI young patients experienced more frequent early recurrence (**Figure 2**).

### Genetic and epigenetic characteristics of sporadic EOCRC

Mutational profiles of *KRAS*, *BRAF* and *TP53,* and the CIMP status were established for all patients (**Table 2**). *PI3KCA* genotyping was performed for 74 tumors. The mutational profile of sporadic EOCRC tumors was similar to that of MSS tumors from old patients for *KRAS*, *TP53* and *PIK3CA* genes, but was clearly different for *BRAF* mutations and CIMP profiles. Sporadic EOCRC tumors neither contained *BRAF* mutations (p = 0.022) nor displayed a CIMP+ profile (p = 0.005) in comparison to tumors from MSS old patients. MSI tumors in old patients had a high rate of *BRAF* mutation (36%) and frequently displayed a CIMP+ profile (62%). MSI young patients differed from MSI old patients by the absence of a methylator phenotype (p = 0.006) and *BRAF* mutations.

**Table 2:** Epigenetic (CIMP status) and genetic alteration profiles for *KRAS*, *BRAF*, *TP53* and *PIK3CA* in the four patient groups.

	MSS young n = 39	MSS old n = 36	MSI young n = 9	MSI old n = 14	p value	p value MSS	p value MSI
**KRAS**					0.77	0.64	1
mutated	14 (37%)	16 (44%)	3 (33%)	4 (29%)			
wild-type	24 (63%)	20 (56%)	6 (67%)	10 (71%)			
***BRAF V600E***					0.001	0.022	0.12
mutated	0 (0%)	5 (14%)	0 (0%)	5 (36%)			
wild-type	39 (100%)	31 (86%)	9 (100%)	9 (64%)			
***TP53***					0.049	0.82	0.12
mutated	17 (44%)	17 (47%)	0 (0%)	5 (36%)			
wild-type	22 (56%)	19 (53%)	9 (100%)	9 (64%)			
***PIK3CA***					0.001	0.11	0.11
mutated	5 (21%)	2 (6%)	4 (80%)	3 (27%)			
wild type	19 (79%)	32 (94%)	1 (20%)	8 (73%)			
**CIMP**					1.9 e^-06^	0.005	0.006
unmethylated	38 (100%)	29 (81%)	9 (100%)	5 (38%)			
methylated	0 (0%)	7 (19%)	0 (0%)	8 (62%)			

Values are expressed as absolute numbers with their relative percentages. M: mutated. WT: wild-type. CIMP: CpG Island Methylator Phenotype.

### Transcriptome analysis

**Figure 3:**
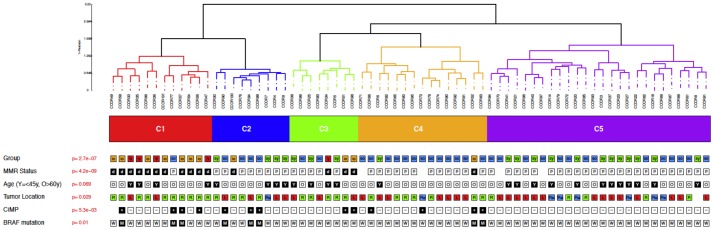
Unsupervised analysis of expression profiles of the four patient groups. Unsupervised classification of expression profiles: dendrogram of the closest clustering to the consensus partition for k = 5. Legend: io, MSI/Old; iy, MSI/Young; so, MSS/Old; sy, MSS/Young; d, deficient MMR; p, proficient MMR; R, right colon; L, left colon; Re, rectum; M, mutated; W, wild-type.

Microarray data were obtained for 70 patients. Unsupervised analyses revealed that, irrespective of the number of clusters and the percentage of variant genes used, the underlying partitions were consistently and strongly associated with MMR status and groups. Other variables significantly associated with the unsupervised partitions were tumor location, CIMP status and mutated BRAF. These variables were themselves closely associated with MMR status. Age was slightly associated with some unsupervised partitions (obtained using 5% and 20% of the most varying genes). The consensus partition in the 5 clusters was most closely associated with the four groups defined on MMR status and age (**Figure 3**). Cluster 1 (C1) was almost exclusively composed of deficient MMR tumors. Cluster C2 was enriched with proficient MMR and BRAF-mutated tumors. Clusters C3 and C4 contained more CIMP+, C4 being almost exclusively composed of proficient MMR tumors from old patients. Cluster 5 (C5) was enriched in sporadic EOCRC tumors found in the left colon.

**Figure 4:**
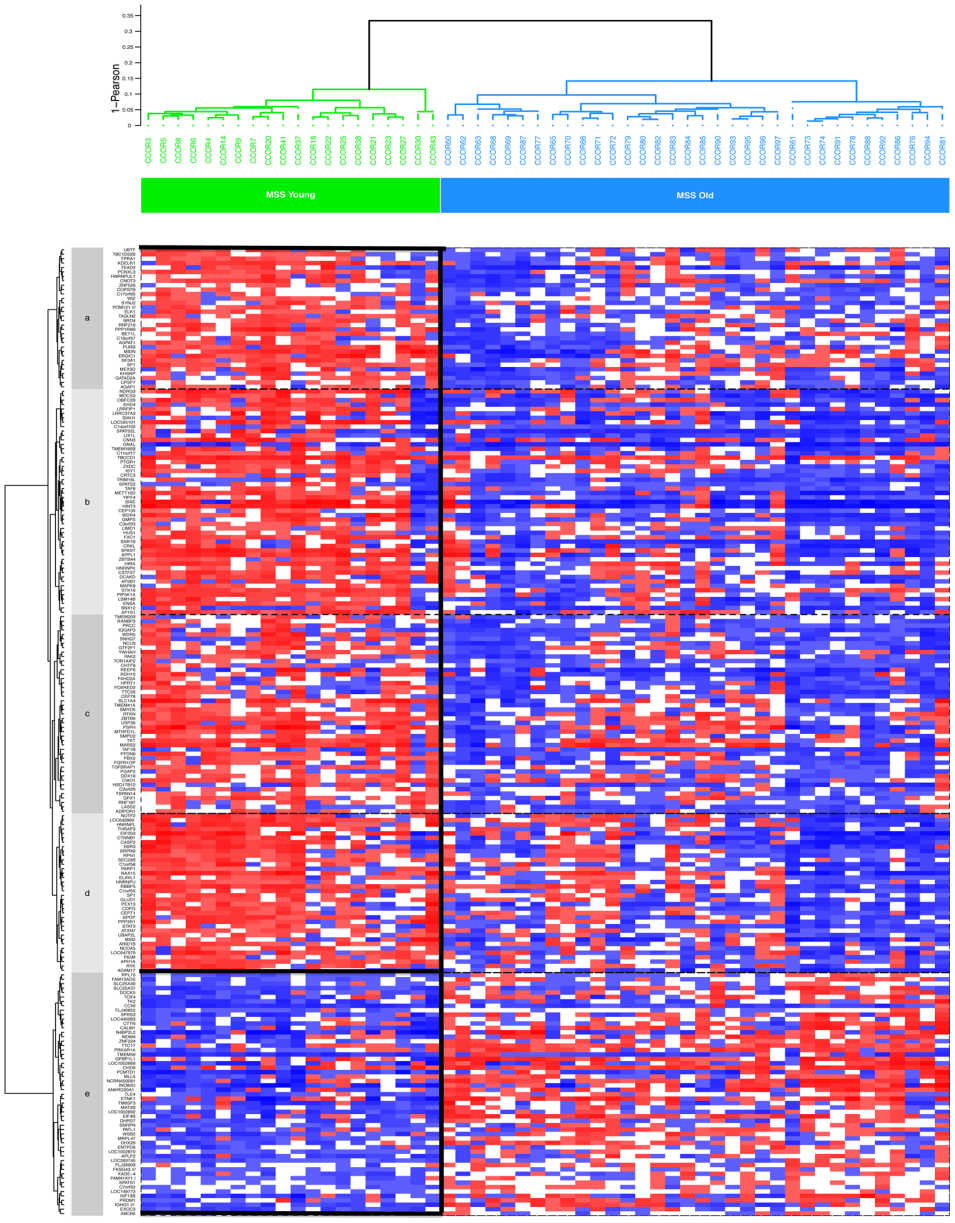
Supervised analysis of MSS tumors (n = 54). The heat map shows the expression of 219 genes. Based on expression levels, genes were grouped into four clusters of highly overexpressed genes (red boxes surrounded by a black border) and one cluster of underexpressed genes in MSS tumors (blue boxes surrounded by a black border) from young patients compared to MSS tumors from older patients. MSS: Microsatellite Stable. MSI: Microsatellite Instability.

**Figure 5:**
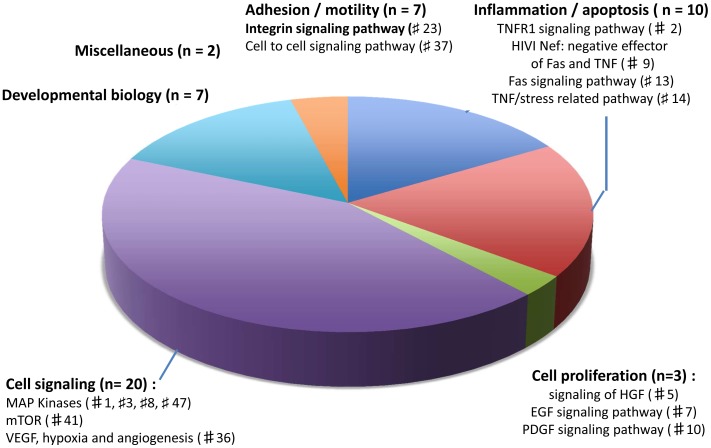
Pathway analysis of MSS tumors. Pathway analysis grouping the 49 most significantly enriched pathways for differential gene expression between sporadic EOCRC and MSS tumors from old patients. Pathways were grouped into five main categories, namely cell adhesion/motility, inflammation/apoptosis, cell proliferation, cell signalling and developmental biology. The distribution of the main canonical pathways among categories is detailed as well as their rank order. This rank order is indicated by the # symbol followed by the row number (# 1 means that this pathway is the most deregulated among the 49 pathways).

We next conducted a supervised analysis of MSS tumors (n = 54) that provided a list of 297 probe sets accounting for 219 highly discriminatory genes (p<0.001). This set provided a clear distinction between sporadic EOCRC tumors and tumors from old MSS patients (**Figure 4**). An extensive description of these genes is provided in **Table S2**. The only key CRC oncogenesis gene that was highly deregulated in sporadic EOCRC was beta catenin (*CTNNB1*). The high number of discriminatory genes allowed us to carry out pathway analyses to better understand sporadic EOCRC carcinogenesis. Forty-nine pathways were significantly enriched for differential gene expression in sporadic EOCRC compared to MSS tumors from old patients. A full description of them is provided in **Table S3**. Among them, 20 were involved in cell signaling, 10 in inflammation and apoptosis, 7 in adhesion and/or motility, 7 in developmental biology and 3 in cell proliferation (**Figure 5**). Several pathways which were up-regulated in sporadic EOCRC were related to major signaling pathways involved in CRC such as MAP kinase and PI3KCA/AKT or are related to growth factors (EGF, HGFR and PDGF) and angiogenesis (VEGF). Two major pathways involved in adhesion and/or motility regulation (integrin signaling pathways and cell-to-cell adhesion signaling) were up-regulated in sporadic EOCRC. Interestingly, most of the pathways related to inflammation and/or apoptosis that were found up regulated in sporadic EOCRC are directly or indirectly linked to the TNF-R1 pathway (TNFR1 pathway, HIV-1Nef, FAS signaling pathway, TNF/Stress Related signaling).

### Beta catenin immunohistochemistry

**Figure 6:**
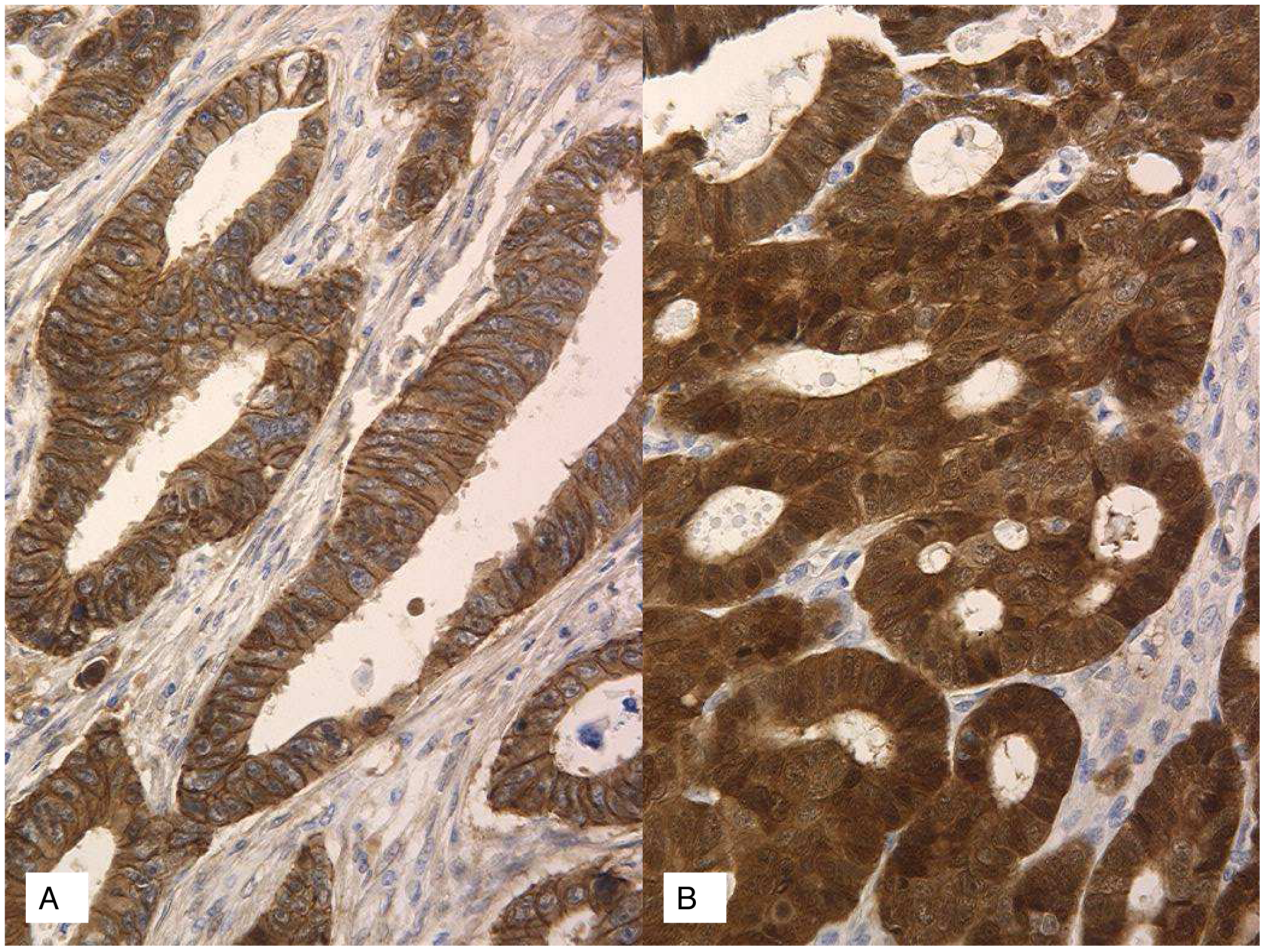
Beta catenin immunostaining in MSS tumors. **A.** Cross-section of a tumor from an MSS-Old patient shows only membrane staining. **B**. Cross-section of a tumor from a sporadic EOCRC (MSS–Young) patient shows strong cytoplasmic and nuclear staining with a loss of membrane staining, reflecting Wnt/beta catenin activation.

The microarray data above identified beta catenin to be the only key CRC oncogenesis gene deregulated in sporadic EOCRC. We therefore sought to assess beta catenin activation using immunohistochemistry. All cases of MSS tumors from young patients (39 cases) and 33 cases (33/36) of MSS tumors from old patients were validated for beta catenin activation using immunohistochemistry. Beta catenin activation was identified in 17 cases (44%) from MSS-young patients and 5 cases (15%) from MSS-old patients (p = 0.01) with a nuclear +/- cytoplasmic staining (**Figure 6**).

## Discussion

The results of the present study suggest that sporadic EOCRC is a distinct clinical and molecular entity. For the first time we provide an integrated analysis of both extensive clinical data and genomics from a carefully screened series of consecutive patients who had been treated and followed-up at a single institution. This translational approach has allowed us to refine the previously limited description of this specific form of CRC and shed insight into its specific tumor biology.

At the moment there is no accepted clear definition of early age of onset in the field of CRC. Therefore, most studies reporting on this topic include patients in the 40–50 years of age range [22], [23]. However, there is a progressive rise in the frequency of CRC between 40 and 50 years of age so it is likely that in these studies there is a mix of specific EOCRC entity and late onset CRC. To address this issue we used a more restrictive definition of both early and late onset CRC: patients between 45 and 60 years of age were excluded in order to ensure a clear partition of the two entities and avoid any overlap of these two forms of CRC. Another confusing factor in the analysis of EOCRC in many studies is the lack of distinction between the hereditary forms of CRC, particularly Lynch syndrome, and the true sporadic form of CRC. However, Lynch syndrome itself accounts for one-third of EOCRC cases [24]. Therefore, in the present study a full pedigree was established by a clinical geneticist for all young patients. Lynch syndrome cases were identified using robust criteria and were clearly separated from sporadic EOCRC. Despite this, in this study sporadic EOCRC patients had a high rate of family history (38%). A minority of them presented with an autosomal dominant transmission inheritance (8%) that probably corresponds to a hereditary disease for which gene susceptibility has not yet been identified (X syndrome). On the other hand, it is increasingly recognized that a few minor predisposition loci could be responsible for a complex form of CRC heredity [25]. Therefore, such a genetic predisposition could also be involved in some patients with EOCRC. This hypothesis is supported by the low rate of synchronous adenoma we observed in sporadic EOCRC patients which could reflect accelerated carcinogenesis secondary to predisposing conditions.

It has long been acknowledged that the morphological specificities of EOCRC are poor cell differentiation, colloid component and lymphocytic stroma reaction [22], [26]–[29]. Again, these data are likely confounded by the inclusion of Lynch syndrome patients, which typically are associated with these histological features. In the only two studies which have specifically analyzed MSS-young patients (one with 24 and one with 55 cases), there was no association between mucinous histology, poor differentiation, tumor-infiltrating lymphocytes and early-onset of disease [30], [31]. Our study has confirmed these data and we furthermore show that poor cell differentiation and a mucinous component are clearly associated with MSI status irrespective of age of patient. In contrast, we did not observe an association between lymphatic invasion, perineural invasion or signet ring cell histology with sporadic EOCRC, as was reported in the two previous studies mentioned above (data not shown) [30], [31].

From a clinical point of view, our results agree with the clinical description of sporadic EOCRC recently published by Chang et al.: Sporadic EOCRCs present as tumors with distal location and frequent metastatic disease [30]. In the present study, the strikingly high rate of synchronous metastatic disease in the EOCRC group (41%) led us to match MSS old patients for tumor stage so as not to bias gene expression comparisons. Although not statistically significant, young patients tended to have better survival rates according to the stage of disease in comparison with older patients. Of course, younger patients are likely to undergo more aggressive therapy including chemotherapy or repeated surgical resection for metastatic disease that may improve the poorer prognosis given for EOCRC. Although partly contradictory with older studies, some of which were global analyses based on registries, the present work provides reliable results for the same aforementioned reasons [32]–[35]. Overall, sporadic EOCRC presents as an aggressive disease with distal location, high tumor stage and no clear histological specificity.

The present study is the first to provide an overview of the frequency of a standard panel of gene mutations as well as epigenetic alterations specifically for sporadic EOCRC. Established colorectal carcinogenesis classifications, using a combination of KRAS and BRAF mutations with CIMP and MSI status, allow the identification of three to five distinct molecular sub-groups of CRC [36], [37]. For example, KRAS and P53 mutations are classically associated with CIN-colorectal cancers whereas BRAF mutations are strongly associated with sporadic forms of CIMP+/MSI+ CRC. A few studies have investigated the mutation rates in sporadic EOCRC cases and shown that 0 to 8% have *BRAF* mutations, 0 to 4% *PIK3CA* mutations, 6 to 78% *KRAS* mutations, and 64% *TP53* mutations [30],[31],[37]-[40]. We identified a rate for *KRAS* and *TP53* mutations (respectively 37% and 44%) as similar to that of CIN tumors but with an absence of *BRAF* mutations and no evidence of a methylator phenotype [41]. The discrepancies with data from the literature on mutation rates in EOCRC might be linked to the absence of a clear distinction between MSS and MSI tumors in older studies which may have affected the mutation rates, particularly for *KRAS* and *BRAF* [39], [40]. Overall, our genetic study favors the hypothesis that sporadic EOCRC is a sub-group of CIN tumors with neither *BRAF* mutation nor methylator phenotype.

The clustering obtained by unsupervised analyses showed that differences in gene expression profiles (GEP) were mainly determined by MMR status and to a lesser extent by CIMP status, while age had little impact. These results have previously been demonstrated by studies showing that MSI and MSS tumors have differential expression profiles, which validates the present results [42]. However, although GEPs were highly significant in supervised analyses restricted to MSS tumors, showing a set of 219 genes differentiating young from old patients, the results of expression arrays must be considered with caution since gene lists frequently fail to be reproduced. In contrast, pathway analyses have proved to be reproducible, and bring biological meaning to gene expression arrays [43]. Numerous pathways classically involved in CRC oncogenesis were up-regulated in sporadic EOCRC, such as MAP-Kinase, mTOR and VEGF/angiogenesis, but pathways related to growth factor signaling are also involved (EGF, HGF receptor and PDGF). We also identified *CTNNB1* as one of the most over-expressed genes in MSS-young patients compared to MSS-old patients and used immunohistochemistry to demonstrate that this leads to an over-activation of beta catenin in sporadic EOCRC. From a treatment perspective, cell signaling activation could indicate sensitivity to inhibitors of these pathways. Anti-EGFR and anti-VEGF antibodies are already used to a large extent to treat metastatic colorectal cancer [44], therefore overexpression of genes from the EGF and VEGF pathways in sporadic EOCRC could improve the response rates to these targeted therapies. Several clinical trials using inhibitors of HGF/Met, MAP-kinase, m-TOR pathways and Wnt-signaling or small-molecule beta catenin inhibitors are ongoing in metastatic colorectal cancer, and optimal patient selection for effective use of these antagonists remains to be established [45]. We also identified two other categories of pathways which were deregulated in sporadic EOCRC. The first one is related to adhesion/motility and is up-regulated, which could reflect the aggressiveness of this tumor type and its high capacity for metastasis. The second one is related to apoptosis and inflammation. In agreement with the previous gene expression profile analysis study of EOCRC, our study also demonstrates the role of inflammation or the immune response in sporadic EOCRC patients compared to late-onset CRC patients [46]. We have identified the TNF-R1 pathways as a key pathway in sporadic EOCRC. This pathway is a major mediator of inflammation, playing a critical role in cell proliferation, differentiation and apoptosis. TNF-α/TNFR-1 signaling has been reported to act as an endogenous tumor promoter for colon carcinogenesis in inflammatory bowel disease (IBD) [45], [47]. Importantly, in the present study IBD patients were excluded by careful clinical screening and the absence of specific histological lesions on resected specimens. This suggests that TNFR-I signalling could have a role in the carcinogenesis of colon cancer independently of chronic inflammation diseases. The question now remains as to the causes of TNFR 1 activation in cancers of young patients. There are in fact two previous studies related to pathway analysis in EOCRC, both were based on a single case series of 12 EOCRC patients [48], [49]. However, their approach was clearly different to this study since they compared gene expression in normal mucosa from EOCRC with colonic mucosa from healthy controls, whereas this study has compared various types of colorectal tumors with each other. The other studies reported a susceptibility gene set for EOCRC that integrated diverse signalling pathways such as focal adhesion and immunosuppression along with epithelial-mesenchymal transition pathways. Although a comparison between these studies and ours should be treated with caution, the disruption of the immune response and the adhesion process seems to play a crucial role both in normal mucosa and EOCRC tumors.

We recently described a molecular classification of colon cancer with prognostic values [10]. This classification identified six molecular types, including three CIN+ CIMP- sub-types: one associated with down-regulated immune pathways, one with up-regulation of the Wnt pathway, and one displaying a normal-like gene expression profile. Our group of sporadic EOCRC, characterized by a CIN-like profile, CIMP-, and beta catenin activation can be compared with the CIN+ subtype of Marisa's classification with an up-regulation of the Wnt pathway, but it also has a specific up-regulation of the TNF-R1 and adhesion/motility pathways. However, such comparisons should be interpreted carefully since they are based on somewhat different pathologies: stage II/II colon cancer excluding rectal cancer for Marisa's classification and predominately stage IV rectal cancer for our classification.

From the data reported here we can conclude that EOCRC arising in patients with no identified genetic predisposition is a specific entity, clearly distinct from the other subgroups of CRC. Clinical specificities are a distal and aggressive disease as well as infrequent synchronous adenomas. The carcinogenesis of this specific tumor type appears to involve a sub-type of the CIN pathway, with no involvement of the methylator pathway and no mutation of *BRAF*. Gene expression profiling has revealed signatures that are specific for sporadic EOCRC. These potentially implicate the TNF-R1 pathway, several other cell signaling pathways and an up-regulation of the Wnt/beta catenin pathway. Further experimental studies are required to decipher the role of such pathways in the development of this particular subtype of CRC. This could be of high relevance to the clinical management of patients since biotherapies such as antibodies are readily available.

## Supporting Information

Table S1 Studied variables. Details of the 79 clinical and tumor characteristics used for statistical analyses.
(DOC)

Table S2 Gene list from the supervised analysis of MSS tumors (n = 54). List of the 219 genes differentially expressed between MSS tumors from young and old patients. Negative values indicate underexpressed genes.
(DOCX)

Table S3 Pathway analysis based on genes differentially expressed between EOCRC and MSS tumors from old patients (p<0.05), combining Globaltest, SAM-GS and Tuckey methods. The p values of each method were converted into ranks and filtered (mean rank<100). *positive values correspond to the proportion of genes significantly overexpressed and negative values to the proportion of genes underexpressed in the pathway.
(DOC)
