# Supplementary material for: Sporadic Early-Onset Colorectal Cancer Is a Specific Sub-Type of Cancer: A Morphological, Molecular and Genetics Study
Source: PLoS One. 2014 Aug 1;9(8):e103159. doi: 10.1371/journal.pone.0103159 (PMC4118858; doi:10.1371/journal.pone.0103159)
Supplement: Table S1 — Studied variables. Details of the 79 clinical and tumor characteristics used for statistical analyses. (DOC) [file pone.0103159.s001.doc]

| **Information Category** | **Variables** | **Legend** |
| --- | --- | --- |
| Sample Information | Sample Tumor type | type of the resected element |
| Patient Information | Birth date | (YYYY/MM/DD) |
| Patient Information | ID patient | especially useful to track dependencies between  different samples coming from the same patient |
| Patient Information | Sex | M=Male; F=Female; |
| Tumor Information | Adenoma::Presence | Adenoma in the resected specimen ; 0=no ; 1=yes |
| Tumor Information | Adenoma::Count | Quantity of Adenoma found |
| Tumor Information | Adenoma::Histological Type | Histological type of adenoma : Villous (0), Tubulous (1),  Tubulo-villous (2), serrated (3), multiple types (4) |
| Tumor Information | Grade::Adenoma::Dysplasia | Dysplasia is classified as : 0=low grade ; 1=high grade ;  2=multiple adenomas with different grade. |
| Tumor Information | Cells::Differentiation | level of cell differentiation; H:high; HM:high to mild ;  M:mild; L:low; U:undifferentiated; |
| Tumor Information | Mucinous component | 0 : absent ; 1 : < 10 % ; 2 : 10 - 50 % ; 3 : > 50 % ;  4 : Yes without precision ; ND : not determined |
| Tumor Information | Invasion::Perineural | Tumoral perineural invasion; 0=absent=no invasion ;  1=present=invasion ; EM=not evaluated |
| Tumor Information | Lympho- vascular invasion | 0=absent ; 1=present ; ND = not determined ; |
| Tumor Information | Location of the primary tumor | details about the location of the primary tumor  left colon, right colon, low rectum, multiple location |
| Tumor Information | Lymph nodes::Local::Number removed | number of lymph nodes removed from the resected  primary tumor. They are not all necessarily metastatic  (see Lymph nodes::Local::Number metastasized for the  number of metastatic lymph nodes among the removed) |
| Tumor Information | Lymphoid Reaction | Lymphoid stromoreaction of the tumor. ;  0=small or absent; 1=moderate; 2=high level with follicles;  EM=not evaluated; |
| Tumor Information | Metastasis::at diagnosis | indicates if a metastasis has been detected at diagnosis  or before the first surgery (pre-operative); 0=no; 1=yes; |
| Tumor Information | Metastasis::Site::Count | Synchronous metastasis at diagnosis; 0=none; 1=single ;  2=multiple; |
| Tumor Information | Metastasis::Bone | Bone metastasis; 0=No; 1=Yes |
| Tumor Information | Metastasis::Brain | Brain metastasis; 0=No; 1=Yes |
| Tumor Information | Metastasis::Liver | liver metastasis; 0=No; 1=Yes |
| Tumor Information | Metastasis::Lung | Lung metastasis; 0=No; 1=Yes |
| Tumor Information | Metastasis::Lymph nodes::Distant | distant lymph nodes metastasis; 0=No; 1=Yes |
| Tumor Information | Metastasis::Lymph nodes::Local::Count | Number of metastasized lymph nodes found on  the resected primary tumor |
| Tumor Information | Metastasis::Ovary | Ovary metastasis; 0=No; 1=Yes |
| Tumor Information | Metastasis::Peritoneum | peritoneum metastasis; 0=No; 1=Yes; |
| Tumor Information | Microsatellite Instability | MSI : Microsatellite Instability; 0=no instability ;  1=low MSI ; 2=instability ; |
| Tumor Information | MMR:: MMR status | MMR :MisMatch Repair function: ; 0 = proficient for MMR  function = expression of protein and no microsatellite  instability ; 1 = Deficient for MMR function = loss of protein  expression and/or MSI. |
| Tumor Information | TNM Stage::Colorectal cancer | Staging method defined by UICC/AJCC, 2002 to describe  the patient classification based on the anatomic extent of  the colorectal cancer. 0 = Stage 0 = Tis N0 M0; 1 = stage 1  = T1-T2 N0 M0; 2 = stage 2 = T3-T4 N0 M0;  3 = stage 3 = T1-T4 N1 M0 -> N2 M0;  4 = stage 4 = any T any N M1; |
| Tumor Information | TNM::M | distant metastasis; MX=Distant metastasis cannot be  assessed; M0=No distant metastasis ;  M1=Distant metastasis; ND= Not recorded/Not known; |
| Tumor Information | TNM::N | regional lymph nodes;  N0=No regional lymph nodes metastasis;  N1= Metastasis in 1 to 3 regional lymph nodes;  N2=Metastasis in more than 4 regional lymph nodes; |
| Tumor Information | TNM::T | primary tumor; COLORECTAL CANCER (2002):  T0=Tis=carcinoma in situ / intraepithelial (within  glandular basement membrane) or invasion of lamina  propria (intramucosal); T1=Tumor invades submucosa;  T2=Tumor invades muscularis propria or pericolic tissue;  T3=Tumor penetrates sub-serosa; T4=Tumor perforates  peritoneum or invades other organs or structures |
| Tumor Information | Tumor::Size from surgery (mm) | histological or pathological size of the tumor in mm; |
| Tumor Information | Tumors::Count (recoded) | Number of tumor(s) in the resected specimen; 0=none;  1=single ; 2= multiple |
| Gene and Protein Screening | IHC::MLH1 | Y=Positive = protein expression;  N = Negative = loss of protein expression;  NA = Non available |
| Gene and Protein Screening | IHC::MSH2 | Y=Positive = protein expression;  N = Negative = loss of protein expression;  NA = Non available |
| Gene and Protein Screening | IHC::MSH6 | Y=Positive = protein expression;  N = Negative = loss of protein expression;  NA = Non available |
| Genetic Alteration & Allelotype | Gene mutation::Functional consequence | functional consequence at a protein level for a gene  mutation. |
| Genetic Alteration & Allelotype | Gene mutation::Status | gene mutation M=mutated ;  WT=wild-type=non mutated |
| Genetic Alteration & Allelotype | MLH1::Germline mutation::Status | M=mutated ; WT = wild-type ; |
| Genetic Alteration & Allelotype | MSH2::Germline mutation::Status | M = mutated ; WT = wild-type |
| Clinical Information | Age at diagnosis (year) | (year); |
| Clinical Information | Amsterdams Criteria | Amsterdams criteria : all of the following must apply for  HNPCC diagnosis : 1/ there are at least three relatives  with an HNPCC-associated cancer (large bowel,  endometrium, small bowel, ureter, or renal pelvis),  2/ At least two successive generations are affected,  3/ At least one person was diagnosed before the age of 50  years, 4/ familial adenomatous polyposis has been excluded.  ; Here, 0 stands for No Amsterdams criteria,  1 for Amsterdams criteria, NA=not relevant and EM=unknown. |
| Clinical Information | Lynch syndrome::Diagnostic | 0 = no = no Lynch diagnosed ; 1=yes= Lynch diagnosed  2=Lynch highly suspected. |
| Clinical Information | Antecedent::Family first-degree | First-degree relatives: mother, father, children and  siblings ; 0=no antecedent ; 1=colonic cancer and/or  endometrial carcinoma ; NA=not relevant; EM=unknown |
| Clinical Information | Antecedent::Family second-degre | Second-degree relatives: grandparents, grandchildren,  aunt, uncle, niece and nephew, half sister and half  brother; 0=no antecedent ; 1=colonic cancer and/or  endometrial carcinoma ; NA=not relevant; EM=unknown |
| Clinical Information | Antecedent::Personal | Personal antecedent; 0=no antecedent ; 1=colonic cancer  and/or endometrial carcinoma ; NA=not relevant;  EM=unknown |
| Clinical Information | Associated Event::Occlusion | 0=no ; 1=yes |
| Clinical Information | Associated Event::Perforation | 0=no ; 1=yes |
| Clinical Information | Diagnosis::Date | Diagnosis date (YYYY/MM/DD) of the primary tumor.  It could be the same as the surgery date or the first  metastasis detection date. |
| Clinical Information | Event::Death (recoded) | patient status at last news time: 0=patient is alive;  1=patient deceased; |
| Clinical Information | Event::Death::Cause | cause of death ; EM = unknown |
| Clinical Information | Event::Death::Date | Date of patient death (YYYY/MM/DD) |
| Clinical Information | Last news::Date | date of the latest medical news concerning the patient  (YYYY/MM/DD); could be last visit/exam date or date of  death; |
| Clinical Information | Relapse::Local | local recurrence; 0=no; 1=yes=local relapse occurred |
| Clinical Information | Relapse::Local::Date | date of local relapse |
| Clinical Information | Relapse::Metastasis | presence of relapse metastasis; 0=absence ;  1=presence ; EM = missing value. |
| Clinical Information | Relapse::Metastasis::Bone | Presence of relapse metastasis in bone; 0=absence ;  1=presence; EM=unknown |
| Clinical Information | Relapse::Metastasis::Bone::Date | date (YYYY/MM/DD) of relapse characterized by bone  metastasis |
| Clinical Information | Relapse::Metastasis::Brain | Presence of relapse metastasis in brain; 0=absence ;  1=presence; EM=unknown |
| Clinical Information | Relapse::Metastasis::Brain::Date | date of metastasis relapse in the brain |
| Clinical Information | Relapse::Metastasis::Lymph Node | Presence of relapse metastasis in ganglion; 0=absence ;  1=presence; EM=unknown |
| Clinical Information | Relapse::Metastasis:: Lymph Node::Date | date of relapse characterized by ganglion metastasis |
| Clinical Information | Relapse::Metastasis::Liver | Presence of relapse metastasis in liver; 0=absence ;  1=presence; EM=unknown |
| Clinical Information | Relapse::Metastasis::Liver::Date | date (YYYY/MM/DD) of relapse characterized by liver  metastasis |
| Clinical Information | Relapse::Metastasis::Lung | Presence of relapse metastasis in lung; 0=absence ;  1=presence; EM=unknown |
| Clinical Information | Relapse::Metastasis::Lung::Date | date of relapse characterized by lung metastasis  (YYYY/MM/DD) |
| Clinical Information | Relapse::Metastasis::Ovary | indiactes if a metastatic relapse has been detected in the  ovary. 0=no ; 1=yes. |
| Clinical Information | Relapse::Metastasis::Ovary::Date | date of the metastatic relapse in the ovary  (YYYY/MM/DD) |
| Clinical Information | Relapse::Metastasis::Peritoneum | relapse metastasis in peritoneum ; 0=absence ;  1=presence ; EM = unknown |
| Clinical Information | Relapse::Metastasis::Peritoneum::Date | date of the metastatic relapse in the peritoneum  (YYYY/MM/DD) |
| Clinical Information | Relapse::Metastasis::Site::Count | Relapse metastatis; 1=single ; 2=multiple ;  EM = unknown |
| Clinical Information | Second cancer::Metachrone carcinoma | 0=absence ; 1=presence ; EM=unknown |
| Clinical Information | Second cancer::Metachrone carcinoma::Date | Date (YYYY/MM/DD) of the metachrone carcinoma. |
| Clinical Information | Surgery::At Diagnosis::Resection quality | This indicates the residual tumor after resection.  R0=no tumor residue ; R1=microscopic residue ;  R2=macroscopic residue ; EM = unknown |
| Clinical Information | Surgery::Extent | extent of surgery; complete or partial resection,  or biopsy; |
| Clinical Information | Surgery::First::Date | Date (YYYY/MM/DD) of curative surgery of the primary  tumor. It might be the same as the diagnostic date but  could also be after (especially if neoadjuvant treatment  have been performed) |
| Clinical Information | Surgery::First::Performed | Y=Yes=surgery performed for the primary tumor;  N=No=no surgery performed for the primary tumor. |
| Other Information | Groups: groups of patients | 1 = MSS- tumor developed under 45 yrs  2= MSS-tumor developed over 60 yrs ,  3= MSI-tumor developed under 45 yrs  4 = MSI-tumor developed over 60 yrs |

**Additional Table 1:** Studied variables. Details of the 79 clinical and tumor characteristics used for statistical analysis.
