# Supplementary material for: Sporadic Early-Onset Colorectal Cancer Is a Specific Sub-Type of Cancer: A Morphological, Molecular and Genetics Study
Source: PLoS One. 2014 Aug 1;9(8):e103159. doi: 10.1371/journal.pone.0103159 (PMC4118858; doi:10.1371/journal.pone.0103159)
Supplement: Table S2 — Gene list from the supervised analysis of MSS tumors (n = 54). List of the 219 genes differentially expressed between MSS tumors from young and old patients. Negative values indicate underexpressed genes. (DOCX) [file pone.0103159.s002.docx]

| **Gene.Symbol** | **Gene.Title** | **adj.P.Val** | **logFC** |
| --- | --- | --- | --- |
| SIAE | sialic acid acetylesterase | 4,2E-05 | 1,95 |
| EHD4 | EH-domain containing 4 | 5,0E-04 | 0,78 |
| HINT3 | histidine triad nucleotide binding protein 3 | 5,0E-04 | 2,82 |
| APH1A | anterior pharynx defective 1 homolog A (C. elegans) | 5,1E-04 | 0,58 |
| EIF4B | eukaryotic translation initiation factor 4B | 5,1E-04 | -0,78 |
| PIP5K1A | phosphatidylinositol-4-phosphate 5-kinase, type I, alpha | 5,1E-04 | 0,87 |
| FXC1 | fracture callus 1 homolog (rat) | 9,0E-04 | 0,57 |
| CASP2 | caspase 2, apoptosis-related cysteine peptidase | 9,4E-04 | 1,31 |
| CTNNB1 | catenin (cadherin-associated protein), beta 1, 88kDa | 9,4E-04 | 1,22 |
| AGPAT1 | 1-acylglycerol-3-phosphate O-acyltransferase 1 (lysophosphatidic acid acyltransferase, alpha) | 2,0E-03 | 0,58 |
| APPL1 | adaptor protein, phosphotyrosine interaction, PH domain and leucine zipper containing 1 | 2,0E-03 | 1,07 |
| COPG | coatomer protein complex, subunit gamma | 2,0E-03 | 0,59 |
| COPS7B | COP9 constitutive photomorphogenic homolog subunit 7B (Arabidopsis) | 2,0E-03 | 0,32 |
| GMPS | guanine monphosphate synthetase | 2,0E-03 | 0,69 |
| LSM14B | LSM14B, SCD6 homolog B (S. cerevisiae) | 2,0E-03 | 0,70 |
| N4BP2L2 | NEDD4 binding protein 2-like 2 | 2,0E-03 | -1,17 |
| PEX13 | peroxisomal biogenesis factor 13 | 2,0E-03 | 0,80 |
| STAT3 | signal transducer and activator of transcription 3 (acute-phase response factor) | 2,1E-03 | 0,52 |
| WDR4 | WD repeat domain 4 | 2,1E-03 | 0,90 |
| SAR1B | SAR1 homolog B (S. cerevisiae) | 2,7E-03 | 0,74 |
| SPOP | speckle-type POZ protein | 3,0E-03 | 0,67 |
| WDR5 | WD repeat domain 5 | 3,0E-03 | 0,58 |
| CSTF2T | cleavage stimulation factor, 3' pre-RNA, subunit 2, 64kDa, tau variant | 3,3E-03 | 0,60 |
| PURB | purine-rich element binding protein B | 3,4E-03 | 0,61 |
| INO80D | INO80 complex subunit D | 3,7E-03 | -1,12 |
| THRAP3 | thyroid hormone receptor associated protein 3 | 3,8E-03 | 0,49 |
| ADIPOR1 | adiponectin receptor 1 | 3,9E-03 | 0,39 |
| HNRNPL | heterogeneous nuclear ribonucleoprotein L | 3,9E-03 | 0,80 |
| SPATS2L | spermatogenesis associated, serine-rich 2-like | 4,1E-03 | 0,75 |
| ZBTB44 | zinc finger and BTB domain containing 44 | 4,1E-03 | 0,35 |
| FAM13AOS | FAM13A opposite strand (non-protein coding) | 4,3E-03 | -0,34 |
| SFRS3 | Splicing factor, arginine/serine-rich 3 | 5,2E-03 | -0,64 |
| CALM1 | Calmodulin 1 (phosphorylase kinase, delta) | 5,3E-03 | -0,70 |
| DCAKD | dephospho-CoA kinase domain containing | 5,5E-03 | 0,64 |
| YIPF4 | Yip1 domain family, member 4 | 6,1E-03 | 0,50 |
| GATAD2A | GATA zinc finger domain containing 2A | 6,1E-03 | 0,48 |
| SLC25A37 | Solute carrier family 25, member 37 | 6,3E-03 | -0,56 |
| METT10D | methyltransferase 10 domain containing | 6,4E-03 | 0,91 |
| RDH10 | retinol dehydrogenase 10 (all-trans) | 7,6E-03 | 0,71 |
| RANBP3 | RAN binding protein 3 | 7,9E-03 | 0,41 |
| C1orf58 | chromosome 1 open reading frame 58 | 8,3E-03 | 0,66 |
| CPSF7 | cleavage and polyadenylation specific factor 7, 59kDa | 8,3E-03 | 0,28 |
| GTF2F1 | general transcription factor IIF, polypeptide 1, 74kDa | 8,3E-03 | 0,44 |
| MIDN | midnolin | 8,3E-03 | 0,88 |
| TMEM41A | transmembrane protein 41A | 8,4E-03 | 0,47 |
| MOCS3 | molybdenum cofactor synthesis 3 | 9,4E-03 | 0,77 |
| PBX2 | pre-B-cell leukemia homeobox 2 | 9,5E-03 | 0,44 |
| SEC22B | SEC22 vesicle trafficking protein homolog B (S. cerevisiae) (gene/pseudogene) | 9,5E-03 | 0,41 |
| KIF13B | kinesin family member 13B | 1,0E-02 | -0,69 |
| SPAST | spastin | 1,1E-02 | 0,77 |
| EXOC3 | exocyst complex component 3 | 1,1E-02 | -0,37 |
| SIAH1 | seven in absentia homolog 1 (Drosophila) | 1,1E-02 | 0,71 |
| MDM4 | Mdm4 p53 binding protein homolog (mouse) | 1,1E-02 | -0,60 |
| WSB2 | WD repeat and SOCS box-containing 2 | 1,1E-02 | -0,50 |
| APLP2 | Amyloid beta (A4) precursor-like protein 2 | 1,2E-02 | -0,45 |
| ELK1 | ELK1, member of ETS oncogene family | 1,2E-02 | 0,47 |
| CCNI | Cyclin I | 1,2E-02 | -0,44 |
| PCNXL3 | pecanex-like 3 (Drosophila) | 1,2E-02 | 0,35 |
| PCMTD1 | protein-L-isoaspartate (D-aspartate) O-methyltransferase domain containing 1 | 1,3E-02 | -0,77 |
| RPL10 | ribosomal protein L10 | 1,3E-02 | -0,23 |
| UBAP2L | ubiquitin associated protein 2-like | 1,3E-02 | 0,36 |
| ZNF224 | Zinc finger protein 224 | 1,3E-02 | -0,41 |
| NAA15 | N(alpha)-acetyltransferase 15, NatA auxiliary subunit | 1,3E-02 | 0,96 |
| TBC1D22B | TBC1 domain family, member 22B | 1,3E-02 | 0,33 |
| NUTF2 | nuclear transport factor 2 | 1,3E-02 | 0,67 |
| LOC100289246 | hypothetical protein LOC100289246 | 1,4E-02 | -0,46 |
| KDELR1 | KDEL (Lys-Asp-Glu-Leu) endoplasmic reticulum protein retention receptor 1 | 1,5E-02 | 0,53 |
| CEP135 | centrosomal protein 135kDa | 1,5E-02 | 0,26 |
| POM121 /// POM121C | POM121 membrane glycoprotein (rat) /// POM121 membrane glycoprotein C | 1,6E-02 | 0,38 |
| SNRPN | small nuclear ribonucleoprotein polypeptide N | 1,6E-02 | -0,33 |
| HUS1 | HUS1 checkpoint homolog (S. pombe) | 1,7E-02 | 0,40 |
| PRKAR1A | protein kinase, cAMP-dependent, regulatory, type I, alpha (tissue specific extinguisher 1) | 1,7E-02 | -0,48 |
| PIGM | phosphatidylinositol glycan anchor biosynthesis, class M | 1,8E-02 | 0,39 |
| SRPRB | signal recognition particle receptor, B subunit | 1,8E-02 | 0,58 |
| TTC17 | tetratricopeptide repeat domain 17 | 1,8E-02 | -0,40 |
| BRD4 | bromodomain containing 4 | 1,8E-02 | 0,37 |
| TK2 | Thymidine kinase 2, mitochondrial | 1,9E-02 | -0,54 |
| ETNK1 | Ethanolamine kinase 1 | 1,9E-02 | -0,91 |
| TLE4 | transducin-like enhancer of split 4 (E(sp1) homolog, Drosophila) | 1,9E-02 | -0,29 |
| C1orf55 | chromosome 1 open reading frame 55 | 2,0E-02 | 0,50 |
| DHRS7 | Dehydrogenase/reductase (SDR family) member 7 | 2,0E-02 | -0,37 |
| ERGIC1 | endoplasmic reticulum-golgi intermediate compartment (ERGIC) 1 | 2,0E-02 | 0,76 |
| LASS2 | LAG1 homolog, ceramide synthase 2 | 2,0E-02 | 0,32 |
| RNF187 | ring finger protein 187 | 2,0E-02 | 0,36 |
| RTKN | rhotekin | 2,0E-02 | 0,49 |
| SF3A1 | splicing factor 3a, subunit 1, 120kDa | 2,0E-02 | 0,37 |
| SLC25A30 | Solute carrier family 25, member 30 | 2,0E-02 | -0,23 |
| SSR3 | signal sequence receptor, gamma (translocon-associated protein gamma) | 2,0E-02 | 0,80 |
| TGFBRAP1 | transforming growth factor, beta receptor associated protein 1 | 2,0E-02 | 0,29 |
| TOR1AIP2 | torsin A interacting protein 2 | 2,0E-02 | 0,24 |
| LOC647979 | hypothetical LOC647979 | 2,1E-02 | 0,69 |
| TMEM185B | transmembrane protein 185B (pseudogene) | 2,1E-02 | 0,57 |
| WIZ | widely interspaced zinc finger motifs | 2,1E-02 | 0,23 |
| MRPL47 | mitochondrial ribosomal protein L47 | 2,1E-02 | -0,26 |
| CHTF8 | CTF8, chromosome transmission fidelity factor 8 homolog (S. cerevisiae) | 2,1E-02 | 0,28 |
| ADAM17 | ADAM metallopeptidase domain 17 | 2,2E-02 | 0,61 |
| C14orf102 | chromosome 14 open reading frame 102 | 2,2E-02 | 0,25 |
| ENTPD6 | ectonucleoside triphosphate diphosphohydrolase 6 (putative function) | 2,2E-02 | -0,32 |
| LIX1L | Lix1 homolog (mouse)-like | 2,2E-02 | 0,50 |
| RYK | RYK receptor-like tyrosine kinase | 2,2E-02 | 0,49 |
| SPATS1 | spermatogenesis associated, serine-rich 1 | 2,2E-02 | -0,18 |
| PRCC | papillary renal cell carcinoma (translocation-associated) | 2,2E-02 | 0,33 |
| KHSRP | KH-type splicing regulatory protein | 2,2E-02 | 0,42 |
| MAPK8 | mitogen-activated protein kinase 8 | 2,2E-02 | 0,32 |
| NCRNA00081 | non-protein coding RNA 81 | 2,2E-02 | -0,67 |
| ZBTB9 | zinc finger and BTB domain containing 9 | 2,3E-02 | 0,43 |
| DHX29 | DEAH (Asp-Glu-Ala-His) box polypeptide 29 | 2,3E-02 | -0,46 |
| LOC149773 | hypothetical protein LOC149773 | 2,3E-02 | -0,14 |
| PGAP2 | post-GPI attachment to proteins 2 | 2,3E-02 | 0,40 |
| PSPH | phosphoserine phosphatase | 2,4E-02 | 0,95 |
| CRKL | v-crk sarcoma virus CT10 oncogene homolog (avian)-like | 2,4E-02 | 0,76 |
| EIF2S3 | eukaryotic translation initiation factor 2, subunit 3 gamma, 52kDa | 2,4E-02 | 1,10 |
| HNRNPK | heterogeneous nuclear ribonucleoprotein K | 2,4E-02 | 0,22 |
| YWHAH | tyrosine 3-monooxygenase/tryptophan 5-monooxygenase activation protein, eta polypeptide | 2,4E-02 | 0,46 |
| C17orf65 | chromosome 17 open reading frame 65 | 2,5E-02 | 0,34 |
| C3orf33 | chromosome 3 open reading frame 33 | 2,5E-02 | 0,31 |
| FAM41AY1 /// FAM41AY2 | family with sequence similarity 41, member A, Y-linked 1 /// family with sequence similarity 41, member A, Y-linked 2 | 2,5E-02 | -0,22 |
| FLJ40852 | hypothetical LOC285962 | 2,5E-02 | -0,19 |
| IGHG1 /// LOC100293559 | Immunoglobulin heavy constant gamma 1 (G1m marker) /// Similar to hCG1812074 | 2,5E-02 | -0,52 |
| PARP1 | poly (ADP-ribose) polymerase 1 | 2,5E-02 | 0,38 |
| PPP1R9B | protein phosphatase 1, regulatory (inhibitor) subunit 9B | 2,5E-02 | 0,61 |
| RPN1 | ribophorin I | 2,5E-02 | 0,41 |
| STK16 | serine/threonine kinase 16 | 2,5E-02 | 0,47 |
| NDRG3 | NDRG family member 3 | 2,6E-02 | 0,96 |
| GNAL | guanine nucleotide binding protein (G protein), alpha activating activity polypeptide, olfactory type | 2,6E-02 | 0,71 |
| SNX12 | sorting nexin 12 | 2,6E-02 | 0,51 |
| PTGR1 | Prostaglandin reductase 1 | 2,7E-02 | 0,50 |
| RNF216 | ring finger protein 216 | 2,7E-02 | 0,41 |
| TMEM59 | transmembrane protein 59 | 2,7E-02 | -0,54 |
| MEX3D | mex-3 homolog D (C. elegans) | 2,7E-02 | 0,66 |
| LIMD1 | LIM domains containing 1 | 2,8E-02 | 0,48 |
| LOC642869 /// SET | SET translocation (myeloid leukemia-associated) pseudogene /// SET nuclear oncogene | 2,8E-02 | 0,63 |
| SYNJ2 | synaptojanin 2 | 2,9E-02 | 0,40 |
| AP1S1 | adaptor-related protein complex 1, sigma 1 subunit | 2,9E-02 | 0,62 |
| FLJ39609 | Similar to hCG1995469 | 3,1E-02 | -0,28 |
| GPBP1L1 | GC-rich promoter binding protein 1-like 1 | 3,1E-02 | -0,57 |
| TKT | transketolase | 3,1E-02 | 0,53 |
| PPP3R1 | protein phosphatase 3, regulatory subunit B, alpha | 3,2E-02 | 0,69 |
| C7orf52 | chromosome 7 open reading frame 52 | 3,3E-02 | -0,23 |
| SLC1A4 | solute carrier family 1 (glutamate/neutral amino acid transporter), member 4 | 3,3E-02 | 0,77 |
| TTC26 | tetratricopeptide repeat domain 26 | 3,3E-02 | 0,46 |
| C11orf17 | chromosome 11 open reading frame 17 | 3,3E-02 | 0,50 |
| CEP78 | centrosomal protein 78kDa | 3,4E-02 | 0,39 |
| HNRNPUL1 | heterogeneous nuclear ribonucleoprotein U-like 1 | 3,4E-02 | 0,49 |
| RBBP5 | retinoblastoma binding protein 5 | 3,4E-02 | 0,65 |
| SMYD5 | SMYD family member 5 | 3,4E-02 | 0,36 |
| ATXN7 | ataxin 7 | 3,4E-02 | 0,38 |
| TAF1B | TATA box binding protein (TBP)-associated factor, RNA polymerase I, B, 63kDa | 3,4E-02 | 0,36 |
| TAGLN2 | transgelin 2 | 3,4E-02 | 0,34 |
| TPRA1 | transmembrane protein, adipocyte asscociated 1 | 3,5E-02 | 0,29 |
| AP2B1 | adaptor-related protein complex 2, beta 1 subunit | 3,5E-02 | 0,57 |
| OBFC2B | oligonucleotide/oligosaccharide-binding fold containing 2B | 3,5E-02 | 0,62 |
| LOC100286909 | Hypothetical protein LOC100286909 | 3,6E-02 | -0,83 |
| HSD17B12 | hydroxysteroid (17-beta) dehydrogenase 12 | 3,6E-02 | 0,55 |
| LOC440983 | hypothetical gene supported by BC066916 | 3,7E-02 | -0,32 |
| DDX18 | DEAD (Asp-Glu-Ala-Asp) box polypeptide 18 | 3,7E-02 | 0,32 |
| ENSA | endosulfine alpha | 3,7E-02 | 0,50 |
| FGFR1OP | FGFR1 oncogene partner | 3,7E-02 | 0,51 |
| CHD9 | chromodomain helicase DNA binding protein 9 | 3,7E-02 | -0,50 |
| TM9SF3 | Transmembrane 9 superfamily member 3 | 3,8E-02 | -0,58 |
| LOC100287025 | hypothetical protein LOC100287025 | 3,8E-02 | -0,21 |
| ZNF526 | zinc finger protein 526 | 3,8E-02 | 0,34 |
| TSPAN14 | tetraspanin 14 | 3,8E-02 | 0,30 |
| TOX4 | TOX high mobility group box family member 4 | 3,8E-02 | -0,50 |
| HPRT1 | hypoxanthine phosphoribosyltransferase 1 | 3,9E-02 | 0,46 |
| CTTN | Cortactin | 3,9E-02 | -0,55 |
| LRRFIP1 | Leucine rich repeat (in FLII) interacting protein 1 | 3,9E-02 | 0,70 |
| FKSG43 /// FRMD8 | FKSG43 gene /// FERM domain containing 8 | 4,0E-02 | -0,19 |
| ISY1 | ISY1 splicing factor homolog (S. cerevisiae) | 4,0E-02 | 0,37 |
| MAT2B | Methionine adenosyltransferase II, beta | 4,0E-02 | -0,30 |
| MSI2 | musashi homolog 2 (Drosophila) | 4,0E-02 | 0,72 |
| SF1 | splicing factor 1 | 4,0E-02 | 0,30 |
| XAGE-4 | XAGE-4 protein | 4,0E-02 | -0,30 |
| SNHG7 | small nucleolar RNA host gene 7 (non-protein coding) | 4,0E-02 | 0,41 |
| FOXRED2 | FAD-dependent oxidoreductase domain containing 2 | 4,1E-02 | 0,45 |
| ANKRD20A1 /// ANKRD20A2 /// ANKRD20A3 /// ANKRD20A4 /// ANKRD20A5 /// C21orf81 /// LOC100132733 /// LOC284232 /// LOC644339 | ankyrin repeat domain 20 family, member A1 /// ankyrin repeat domain 20 family, member A2 /// ankyrin repeat domain 20 family, member A3 /// ankyrin repeat domain 20 family, member A4 /// ankyrin repeat domain 20 family, member A5 /// ankyrin repeat domain 20 family, member A3 pseudogene /// similar to FLJ00310 protein /// ankyrin repeat domain 20 family, member A2 pseudogene /// similar to ANKRD20A2 protein | 4,1E-02 | -0,63 |
| LOC595101 | PI-3-kinase-related kinase SMG-1 pseudogene | 4,1E-02 | 0,77 |
| TRIM16L | tripartite motif-containing 16-like | 4,2E-02 | 0,52 |
| PATL1 | protein associated with topoisomerase II homolog 1 (yeast) | 4,2E-02 | -0,36 |
| LRRC37A3 | leucine rich repeat containing 37, member A3 | 4,3E-02 | 0,76 |
| TAF8 | TAF8 RNA polymerase II, TATA box binding protein (TBP)-associated factor, 43kDa | 4,3E-02 | 0,35 |
| CIAO1 | cytosolic iron-sulfur protein assembly 1 homolog (S. cerevisiae) | 4,3E-02 | 0,35 |
| ELAVL1 | ELAV (embryonic lethal, abnormal vision, Drosophila)-like 1 (Hu antigen R) | 4,3E-02 | 0,30 |
| ARID1B | AT rich interactive domain 1B (SWI1-like) | 4,5E-02 | 0,48 |
| NCLN | nicalin homolog (zebrafish) | 4,5E-02 | 0,48 |
| ZXDC | ZXD family zinc finger C | 4,5E-02 | 0,39 |
| GLUD1 | glutamate dehydrogenase 1 | 4,5E-02 | 0,42 |
| MTHFD1L | methylenetetrahydrofolate dehydrogenase (NADP+ dependent) 1-like | 4,5E-02 | 0,48 |
| HNRNPU | heterogeneous nuclear ribonucleoprotein U (scaffold attachment factor A) | 4,5E-02 | 0,28 |
| REEP6 | receptor accessory protein 6 | 4,5E-02 | 0,73 |
| TBCCD1 | TBCC domain containing 1 | 4,5E-02 | 0,47 |
| SMPD2 | sphingomyelin phosphodiesterase 2, neutral membrane (neutral sphingomyelinase) | 4,5E-02 | 0,39 |
| FAHD2A | fumarylacetoacetate hydrolase domain containing 2A | 4,6E-02 | 0,45 |
| ABCB5 | ATP-binding cassette, sub-family B (MDR/TAP), member 5 | 4,7E-02 | -0,17 |
| CNN3 | calponin 3, acidic | 4,7E-02 | 0,61 |
| DOCK5 | Dedicator of cytokinesis 5 | 4,7E-02 | -0,56 |
| LOC283745 | hypothetical protein LOC283745 | 4,7E-02 | -0,23 |
| 40604 | methionyl-tRNA synthetase 2, mitochondrial | 4,7E-02 | 0,65 |
| PAK2 | p21 protein (Cdc42/Rac)-activated kinase 2 | 4,7E-02 | 0,43 |
| IQGAP3 | IQ motif containing GTPase activating protein 3 | 4,7E-02 | 0,43 |
| USP36 | ubiquitin specific peptidase 36 | 4,8E-02 | 0,44 |
| SPATS2 | spermatogenesis associated, serine-rich 2 | 4,8E-02 | 0,31 |
| NCOA5 | nuclear receptor coactivator 5 | 4,8E-02 | 0,40 |
| PRDM1 | PR domain containing 1, with ZNF domain | 4,8E-02 | -0,39 |
| TEAD2 | TEA domain family member 2 | 4,8E-02 | 0,63 |
| CEPT1 | choline/ethanolamine phosphotransferase 1 | 4,9E-02 | 0,41 |
| C2orf29 | chromosome 2 open reading frame 29 | 4,9E-02 | 0,28 |
| BET1L | blocked early in transport 1 homolog (S. cerevisiae)-like | 4,9E-02 | 0,32 |
| SP1 | Sp1 transcription factor | 4,9E-02 | 0,37 |
| CNOT3 | CCR4-NOT transcription complex, subunit 3 | 4,9E-02 | 0,47 |
| UBTF | upstream binding transcription factor, RNA polymerase I | 4,9E-02 | 0,35 |
| AGAP1 | ArfGAP with GTPase domain, ankyrin repeat and PH domain 1 | 5,0E-02 | 0,42 |
| CRTC3 | CREB regulated transcription coactivator 3 | 5,0E-02 | 0,40 |
| GPX1 | glutathione peroxidase 1 | 5,0E-02 | 0,36 |
| HIRA | HIR histone cell cycle regulation defective homolog A (S. cerevisiae) | 5,0E-02 | 0,60 |
| MLL5 | myeloid/lymphoid or mixed-lineage leukemia 5 (trithorax homolog, Drosophila) | 5,0E-02 | -0,40 |
| PFDN6 | prefoldin subunit 6 | 5,0E-02 | 0,55 |
| TMEM203 | transmembrane protein 203 | 5,0E-02 | 0,28 |
| C16orf57 | chromosome 16 open reading frame 57 | 5,0E-02 | 0,39 |

**Additional Table S2:** Gene list of the supervised analysis of MSS tumors (n=54).

List of the 219 genes differentially expressed between MSS tumors from young and old patients.
