# Supplementary material for: Sporadic Early-Onset Colorectal Cancer Is a Specific Sub-Type of Cancer: A Morphological, Molecular and Genetics Study
Source: PLoS One. 2014 Aug 1;9(8):e103159. doi: 10.1371/journal.pone.0103159 (PMC4118858; doi:10.1371/journal.pone.0103159)
Supplement: Table S3 — Pathway analysis based on genes differentially expressed between EOCRC and MSS tumors from old patients (p<0.05), combining Globaltest, SAM-GS and Tuckey methods. The p values of each method were converted into ranks and filtered (mean rank<100). *positive values correspond to the proportion of genes significantly overexpressed and negative values to the proportion of genes underexpressed in the pathway. (DOC) [file pone.0103159.s003.doc]

| **Pathway name** | **Pathway**  **category** | **Geometric mean of ranks** | **Proportion of genes significantly over/underexpressed in EOCRC*** |
| --- | --- | --- | --- |
| Links between Pyk2 and Map Kinases | Cell Signaling | 10,9 | 60% |
| TNFR1 Signaling Pathway | Apoptosis / inflammation | 14,1 | 85% |
| Angiotensin II mediated activation of JNK Pathway via Pyk2 dependent signaling | Cell Signaling | 16,2 | 56% |
| Agrin in Postsynaptic Differentiation | Developmental biology | 22,1 | 61% |
| Signaling of Hepatocyte Growth Factor Receptor | Cell proliferation | 23,7 | 60% |
| Regulation of PGC-1a | Developmental biology | 25,9 | -80% |
| EGF Signaling Pathway | Cell proliferation | 26,1 | 69% |
| Role of MAL in Rho-Mediated Activation of SRF | Cell Signaling | 31,3 | 82% |
| HIV-I Nef: negative effector of Fas and TNF | Apoptosis / inflammation | 35,3 | 70% |
| PDGF Signaling Pathway | Cell proliferation | 36,8 | 67% |
| Nitric Oxide Signaling Pathway | Cell Signaling | 37,9 | -70% |
| Effects of calcineurin in Keratinocyte Differentiation | Developmental biology | 39,4 | -78% |
| FAS signaling pathway ( CD95 ) | Apoptosis / inflammation | 44,4 | 73% |
| TNF/Stress Related Signaling | Apoptosis / inflammation | 47,4 | 67% |
| Regulation And Function Of ChREBP in Liver | Cell Signaling | 49,1 | 62% |
| Caspase Cascade in Apoptosis | Apoptosis | 49,6 | 67% |
| Bioactive Peptide Induced Signaling Pathway | Cell Signaling | 50,6 | -53% |
| Rho cell motility signaling | Adhesion / motility | 51,0 | 75% |
| Transcription factor CREB and its extracellular signals | Cell Signaling | 54,8 | -77% |
| Signaling Pathway from G-Protein Families | Cell Signaling | 56,5 | -75% |
| BCR Signaling Pathway | Cell Signaling | 57,2 | 57% |
| Activation of cAMP-dependent protein kinase, PKA | Adhesion / motility | 69,6 | -75% |
| Integrin Signaling Pathway | Adhesion / motility | 73,9 | 69% |
| PECAM-1 as a Scaffold for Signaling and Adaptor Molecule | Cell Signaling | 74,1 | 57% |
| Fc Epsilon Receptor I Signaling in Mast Cells | Cell Signaling | 74,8 | 53% |
| Presenilin action in Notch and Wnt signaling | Cell Signaling | 75,8 | 80% |
| Apoptotic Signaling in Response to DNA Damage | Apoptosis / inflammation | 76,0 | 80% |
| Y branching of actin filaments | Adhesion / motility | 76,7 | 60% |
| Regulation of BAD phosphorylation | Apoptosis / inflammation | 79,9 | 50% |
| Transcriptional Activation by ARC Complex | Cell Signaling | 80,3 | 83% |
| Ca++/ Calmodulin-dependent Protein Kinase Activation | Cell Signaling | 80,4 | -86% |
| Inhibition of Cellular Proliferation by Gleevec | Adhesion / motility | 80,5 | 88% |
| Role of MEF2D in T-cell Apoptosis | Apoptosis / inflammation | 82,5 | -56% |
| Erk1/Erk2 Mapk Signaling | Adhesion / motility | 82,8 | 62% |
| Multiple antiapoptotic pathways from IGF-1R signaling lead to BAD phosphorylation | Apoptosis / inflammation | 83,8 | -56% |
| VEGF, Hypoxia, and Angiogenesis | Cell Signaling | 84,4 | 88% |
| Cell to Cell Adhesion Signaling | Adhesion / motility | 85,2 | 71% |
| NFAT and Hypertrophy of the heart (Transcription in the broken heart) | Developmental biology | 86,0 | -56% |
| Nuclear receptors coordinate the activities of chromatin remodeling complexes and coactivators to facilitate initiation of transcription in carcinoma cells | Developmental biology | 88,1 | 57% |
| Endocytotic role of NDK, Phosphins and Dynamin | Miscellaneous | 88,1 | 71% |
| mTOR Signaling Pathway | Cell Signaling | 88,5 | 56% |
| Eukaryotic protein translation | Developmental biology | 89,2 | 80% |
| EPO Signaling Pathway | CellSignaling | 90,8 | 67% |
| Trefoil Factors Initiate Mucosal Healing | Adhesion motility | 92,0 | 56% |
| ChREBP regulation by carbohydrates and cAMP | Miscellaneous | 93,6 | -60% |
| CASK Protein Interactions | CellSignaling | 93,7 | -71% |
| MAPKinase Signaling Pathway | CellSignaling | 95,4 | 57% |
| Regulation of Insulin and IGF Signaling by IRS Proteins | CellSignaling | 97,3 | 50% |
| Control of skeletal myogenesis by HDAC & calcium/calmodulin-dependent kinase (CaMK) | Developmental biology | 99,2 | -60% |

**Additional Table 3:** Pathway analysis based on genes differentially expressed between EOCRC and old MSS patients (p<0.01) combining Globaltest, SAM-GS and Tuckey methods.
